# Teeth

**Published:** 1860-02

**Authors:** 


### TEETH.

We have recently obtained a lot of teeth from the manufacture of Crosby & Co., which possess the points desirable in porcelain teeth, in a very marked degree, viz: form, texture, color, shade, &c.

They may not posses all these points to perfection but they are certainly good teeth. A very considerable discount is made from the ordinary price of such teeth; so much as to be quite an inducement to those who purchase. There are some teeth amongst them unlike anything we have seen before, so that, that far an unoccupied niche is filled by them, and we think it desirable now to have some of them always at hand. T.

